# Acute effects of oral mesna administration on the full amino acid profile and 3-methylhistidine: secondary results from the CYLOB dose-finding study

**DOI:** 10.1007/s00726-024-03398-2

**Published:** 2024-06-06

**Authors:** Thomas Olsen, Amany Elshorbagy, Emma Stolt, Anders Åsberg, Hasse K. Zaré, Nasser E. Bastani, Helga Refsum, Kjetil Retterstøl, Kathrine J. Vinknes

**Affiliations:** 1 Department of Nutrition, Institute of Basic Medical Sciences, Faculty of Medicine, University of Oslo, Oslo, Norway; 2 Department of Pharmacology, University of Oxford, Oxford, UK; 3 Department of Physiology, Faculty of Medicine, University of Alexandria, Alexandria, Egypt; 4 Department of Transplantation Medicine, Oslo University Hospital, Oslo, Norway; 5 Department of Pharmacy, University of Oslo, Oslo, Norway; 6 Department of Pharmacology, Oslo University Hospital, Oslo, Norway; 7 Department of Endocrinology, The Lipid Clinic, Morbid Obesity and Preventive Medicine, Oslo University Hospital, Oslo, Norway

**Keywords:** Mesna, Total cysteine, Overweight, Obesity, Amino acid profile, 3-methylhistidine

## Abstract

Plasma total cysteine (tCys) is strongly associated with fat mass in humans. Mesna lowers plasma tCys in a dose-dependent manner, but it is not known whether it interferes with metabolism of other amino acids or protein. In this Phase-1 study, we show that a single dose of mesna administered at 400, 800, 1200 or 1600 mg to 6–7 individuals per dose only slightly affects amino acid profiles, with increases in plasma valine across dose levels. There were no effects of mesna on 3-methylhistidine, a marker of protein breakdown.

**Supplementary Information:**

The online version contains supplementary material available at 10.1007/s00726-024-03398-2.

## Introduction

Plasma concentrations of total cysteine (tCys) are strongly and linearly associated with overweight and obesity (Elshorbagy et al. 2008, 2012). Dietary restriction of cysteine and its precursor, methionine, leads to significant improvements in body composition of mice (Cooke et al. 2020; Hasek et al. 2013; Malloy et al. 2006, 2013; Nichenametla et al. 2022). Furthermore, in a recent randomized controlled trial (RCT), we substantiated that implementing a dietary restriction of cysteine led to a significantly greater weight loss among human participants (Olsen et al. 2024). This link between cysteine and obesity has motivated interest in the therapeutic potential of pharmacologically lowering plasma tCys concentrations to lower fat mass and obesity in humans. Cysteine-lowering can be achieved with mesna – sodium-2-mercaptoethane sulfonate – a pharmacological thiol agent that forms disulfide bonds with cysteine in the circulation, leading to increased urinary excretion (Lauterburg et al. 1994; Stofer-Vogel et al. 1993; Urquhart et al. 2007). We have previously shown that a single oral dose of mesna up to 1600 mg was well-tolerated and significantly reduced plasma concentrations of tCys (up to 52% reduction at nadir) for up to 12 h post-dose (Vinknes et al. 2023).

Although restricting cysteine availability through the diet distinctly improves body composition in rodents (Dong et al. 2018), it slows rates of protein synthesis in the liver and skeletal muscle (Martinez et al. 2022; Nichenametla et al. 2018), and alters the plasma amino acid pool (Nichenametla et al. 2022). This effect may be due to redirection of amino acids to other metabolic processes, including the tricarboxylic acid (TCA) cycle (Gao et al. 2019), and dietary cysteine restriction may therefore contribute to the observations of lower lean, bone and skeletal muscle mass as compared to controls (Ables et al. 2012; Cooke and Ables 2023; Cooke et al. 2020).

As opposed to dietary cysteine restriction, pharmacological reduction of cysteine using mesna reduce fat mass accumulation but not lean mass accumulation in mice (Vinknes et al. 2023). Taken together with previous evidence (Martinez et al. 2022; Nichenametla et al. 2018; Nichenametla et al. 2022), these demonstrations indicate that the amino acid pool available for protein synthesis may be affected differently in pharmacologic compared to dietary manipulation of cysteine. However, there is no evidence on the effect of mesna on the plasma amino acid pool and/or protein breakdown in humans. To explore this, we utilized data from our previous dose-escalation trial with mesna in humans with overweight and obesity, and investigated whether a single oral dose of mesna (400, 800, 1200 and 1600 mg/d) influenced plasma and urinary excretion of 20 amino acids and 3-methylhistidine, a marker of protein breakdown.

## Methods

The present data are from a phase-1, non-randomized single ascending dose study in healthy volunteer men with overweight and obesity. Details on the study protocol, study design, blood sampling, and main results have been published elsewhere (Vinknes et al. 2023). Briefly, 17 men were assigned to receive 400 mg (*n* = 7), 800 mg (*n* = 6), 1200 mg (*n* = 6) and 1600 mg (*n* = 6) of oral mesna. Blood samples were taken at baseline and 0.5, 1, 1.5, 2, 2.5, 3, 4, 6, 8, 10 and 12 h post-dose into EDTA-lined tubes. Twenty-four-hour urine was collected as described previously (Vinknes et al. 2023). Blood and urine were stored at − 80 ℃ until analysis. Plasma and urine amino acid profiles, and 3-methyl-histidine were measured using liquid chromatography-tandem mass spectrometry using previously published methods (Turner et al. 2023). Measured analytes included alanine, arginine, asparagine, aspartate (plasma only), glutamate, glutamine, glycine, histidine, isoleucine, leucine, lysine, ornithine, phenylalanine, proline, serine, taurine, threonine, tryptophan, tyrosine and valine. All study participants gave written informed consent. The study was performed according to the Declaration of Helsinki, and approved by Regional Ethics Committee South-East (Ref no. 87266).

Area under the plasma concentration versus time curve (AUC) was calculated for each amino acid using the trapezoidal method. P_trend_ was calculated using linear regression in separate models for each amino acid, where the AUC was the dependent variable and the dose was the independent variable. *P*-values < 0.05 were considered statistically significant.

## Results

### Baseline characteristics

Baseline characteristics of the participants has been published previously (Vinknes et al. 2023). Briefly, *n* = 17 men with a mean (SD) age of 43.5 (8.4) years and BMI of 32.7 (3.0) kg/m^2^ participated in the present study. Baseline amino acid profiles according to dose level can be viewed in Supplemental Table S1.

### Plasma and urinary excretion of amino acids by dose level

The AUC for valine increased significantly with increasing dose levels (β = 110 µmol×h/L, 95% CI: 14.6, 205, p_trend_ = 0.03). Similar responses were observed for isoleucine and tryptophan, but these findings did not reach statistical significance (p_trend_ < 0.10, Table 1). No effects of mesna were observed on plasma levels of 3-methylhistidine. Furthermore, no effects of escalating mesna dosing were observed on 24-h urinary excretion of amino acids or 3-methylhistidine (Supplemental Table S2).

**Table 1:** Mean (standard deviation) area under the curve of plasma amino acid concentrations according to mesna dosing

	400 mg	800 mg	1200 mg	1600 mg	β (95% CI)	*P*_trend_
*Amino acid, µmol*×*h/L*						
Alanine	4530 (603)	4000 (794)	4520 (673)	4500 (493)	33.2 (-207, 274)	0.78
Arginine	1130 (172)	1050 (158)	1080 (70.7)	1140 (146)	5.45 (-46.4, 57.3)	0.83
Asparagine	445 (59.6)	429 (84.9)	530 (61.8)	452 (97.5)	12.4 (-17.7, 42.5)	0.40
Aspartate	56.7 (28.9)	43.8 (11.6)	50.1 (8.1)	51.4 (8.44)	-1.20 (-7.5, 5.09)	0.70
Glutamate	758 (274)	563 (172)	641 (214)	745 (123)	0.07 (-78.4, 78.5)	1.00
Glutamine	6800 (874)	6700 (418)	7320 (446)	7160 (638)	165 (-65.1, 396)	0.15
Glycine	2610 (411)	2750 (580)	2650 (541)	2540 (556)	-28.6 (-212, 155)	0.75
Histidine	1050 (94)	1030 (90.9)	1080 (53.9)	1040 (63.8)	1.64 (-26.5, 29.8)	0.91
Isoleucine	924 (120)	1030 (126)	921 (119)	1100 (162)	42.5 (-8.44, 93.4)	0.098
Leucine	1660 (198)	1840 (290)	1650 (132)	1870 (191)	42.9 (-37.2, 123)	0.28
Lysine	2080 (156)	2090 (171)	2120 (169)	2300 (384)	66.5 (-17.7, 151)	0.12
Ornithine	876 (134)	798 (119)	837 (146)	841 (73.3)	-8.05 (-51.7, 35.6)	0.71
Phenylalanine	742 (115)	747 (135)	752 (70.9)	824 (47.9)	24.5 (-10.8, 59.8)	0.16
Proline	2190 (601)	2150 (567)	2990 (1080)	2480 (483)	173 (-97, 443)	0.20
Serine	1120 (114)	1220 (183)	1230 (253)	1160 (248)	16 (-56.8, 88.9)	0.65
Taurine	700 (265)	737 (57.5)	611 (59.8)	725 (113)	-5.13 (-62.9, 52.6)	0.86
Threonine	1490 (157)	1520 (207)	1590 (169)	1500 (302)	11.7 (-64.2, 87.7)	0.75
Tryptophan	301 (64.8)	315 (50.8)	324 (44.1)	350 (33.6)	15.6 (-2.1, 33.2)	0.081
Tyrosine	897 (160)	845 (145)	808 (117)	927 (127)	3.71 (-47.8, 55.3)	0.88
Valine	3230 (265)	3470 (335)	3390 (233)	3620 (200)	110 (14.6, 205)	**0.026**
3-methyl-histidine	151 (71.2)	104 (73.6)	141 (72.8)	120 (43)	-6.07 (-31.6, 19.4)	0.63

## Discussion

In this secondary analysis of a dose-escalation study, we report that administration of increasing single doses of mesna, which effectively lowers plasma tCys (Vinknes et al. 2023), only minimally influences the remaining plasma amino acid pool and 3-methylhistidine. These findings indicate that there are few acute effects of mesna on amino acid metabolism. However, a drawback of the present study is that we were not able to quantify tissue concentrations or flux. In rodents, dietary restriction of cysteine alters plasma concentrations of histidine, phenylalanine, serine, threonine and tryptophan possibly by directing them to the TCA cycle (Gao et al. 2019; Nichenametla et al. 2022), with concomitant lower rates of protein synthesis, reduced growth rates and lower lean mass (Ables et al. 2012; Cooke and Ables 2023; Cooke et al. 2020). Therefore, long-term studies with mesna administration are needed to investigate whether our reported minor effects on the plasma amino acid pool persist over time, and whether there are negative effects on lean mass.

Although we mostly observed neutral effects of a single oral dose of mesna across different dose levels, there were indications of effects on branched-chain amino acids (BCAA). There was a significant increase in valine and a trend for an increase in isoleucine across dose levels. The underlying mechanism is unclear, but is likely indirect considering that mesna works by binding thiols in plasma and facilitate their urinary excretion. Speculatively, our results might relate to the effect of mesna on endogenous cysteine availability. Elimination of dietary cysteine rapidly induces autophagy in multiple experimental model systems (Cooke et al. 2020; Plummer and Johnson 2019; Ruckenstuhl et al. 2014) similar to short-term fasting (Shabkhizan et al. 2023). Notably, during 24 h of fasting in humans, plasma concentrations of BCAAs are elevated by ~ 14 to 38% with only negligible changes in other amino acids (Anfinsen et al. 2023). However, the study in question also reported the largest increase in plasma leucine, which we did not observe after ascending single doses of oral mesna. As such, we cannot exclude the possibility that the observed effect on valine and the weaker effect on isoleucine were due to chance.

This is the first study reporting on the acute effects of mesna on amino acids and 3-methylhistidine in humans with overweight and obesity. It provides preliminary, yet valuable information on whether mesna can affect the metabolism of other amino acids. Our study has clear limitations, including the small sample size, inclusion of men only, the short study duration, and the lack of a control group. In addition, the study was not designed to detect effects of mesna on the plasma or urine amino acid profile. These limitations impede the interpretation and generalizability of our findings. Thus, the results of this study are therefore mainly suitable to inform the planning and design of future studies, where these limitations can be overcome.

In conclusion, the acute effects of mesna on plasma and urinary concentrations of amino acids and 3-methylhistidine are minimal, but experimental and long-term human studies are needed to address whether repeated mesna dosing over time affects amino acid metabolism.

## Electronic supplementary material

Below is the link to the electronic supplementary material.

Supplementary Material 1

## Data Availability

The data are not publicly available due to privacy or ethical restrictions. Data can be requested from the corresponding author but is subject to ethical approvals and institutional collaboration agreements.
