# Supplementary material for: Acute effects of oral mesna administration on the full amino acid profile and 3-methylhistidine: secondary results from the CYLOB dose-finding study
Source: Amino Acids. 2024 Jun 6;56(1):39. doi: 10.1007/s00726-024-03398-2 (PMC11156715; doi:10.1007/s00726-024-03398-2)
Supplement: Supplementary file 1 — Supplementary Material 1 [file 726_2024_3398_MOESM1_ESM.docx]

**Acute effects of oral mesna administration on the full amino acid profile and 3-methylhistidine: Secondary results from the CYLOB dose-finding study**

Thomas Olsen^1,*^, Amany Elshorbagy^2,3^, Emma Stolt^1^, Anders Åsberg^4,5^, Hasse K. Zaré^6^, Nasser E. Bastani^1^, Helga Refsum^1,3^, Kjetil Retterstøl^1,7^, Kathrine J. Vinknes^1^

^1^Department of Nutrition, Institute of Basic Medical Sciences, Faculty of Medicine, University of Oslo, Norway

^2^Department of Pharmacology, University of Oxford, Oxford, United Kingdom
^3^Department of Physiology, Faculty of Medicine, University of Alexandria, Alexandria, Egypt

^4^Department of Transplantation Medicine, Oslo University Hospital, Oslo, Norway
^5^Department of Pharmacy, University of Oslo, Oslo, Norway

^6^Department of Pharmacology, Oslo University Hospital, Oslo, Norway

^5^The Lipid Clinic, Department of Endocrinology, Morbid Obesity and Preventive Medicine, Oslo University Hospital, Oslo, Norway

Correspondence:

Thomas Olsen

[thomas.olsen@medisin.uio.no](mailto:thomas.olsen@medisin.uio.no),

+47-22862585,

Domus Medica, Sognsvannveien 9, 0372 Oslo, Norway

| **Supplemental Table 1.** Baseline median (25^th^, 75^th^ percentile) amino acid concentrations according to mesna dosing | | | | |
| --- | --- | --- | --- | --- |
|  | **400 mg** | **800 mg** | **1200 mg** | **1600 mg** |
| *Amino acid, μmol/L* |  |  |  |  |
| Alanine | 365 (303, 386) | 336 (278, 397) | 353 (277, 389) | 442 (409, 468) |
| Arginine | 75 (71.8, 78.7) | 80.4 (75.9, 89.2) | 88.1 (86.7, 94.3) | 88.6 (85.7, 103) |
| Asparagine | 30.1 (26, 31.8) | 35.2 (26.4, 37.6) | 40.7 (34.2, 46.8) | 34.7 (29.2, 41.2) |
| Aspartate | 3.33 (3.23, 3.71) | 3.66 (3.3, 4.06) | 3.65 (3.47, 5.08) | 3.54 (3.07, 3.99) |
| Glutamate | 62.2 (58.2, 86.1) | 46.5 (37.7, 60.3) | 59.9 (50.4, 66.1) | 55.5 (47.5, 64.7) |
| Glutamine | 533 (516, 574) | 541 (526, 587) | 585 (561, 622) | 592 (565, 639) |
| Glycine | 219 (190, 251) | 237 (210, 278) | 201 (193, 268) | 210 (179, 250) |
| Histidine | 86.6 (81.3, 90) | 80.7 (76.5, 84.5) | 88.7 (84.7, 93.5) | 83.7 (80, 87.2) |
| Isoleucine | 70.6 (69, 82.7) | 78.9 (72.7, 84.4) | 73.6 (67, 80) | 81.7 (76.7, 86.2) |
| Leucine | 137 (129, 147) | 147 (139, 148) | 142 (138, 145) | 146 (142, 151) |
| Lysine | 157 (145, 183) | 166 (154, 179) | 164 (162, 175) | 185 (160, 201) |
| Ornithine | 65.7 (60.2, 78.8) | 59.9 (57.7, 65.5) | 66.7 (57.5, 77.2) | 59.4 (57, 60) |
| Phenylalanine | 56.6 (55.6, 58.1) | 62.8 (54.9, 68.3) | 62.4 (56.9, 68) | 67.2 (63.7, 69.1) |
| Proline | 143 (138, 168) | 151 (145, 192) | 191 (160, 241) | 171 (144, 221) |
| Serine | 91.8 (81.6, 101) | 102 (90.7, 107) | 101 (90.6, 110) | 94.2 (89.6, 105) |
| Taurine | 47.4 (41.4, 54.6) | 58.2 (57.2, 64.4) | 48.1 (45.5, 52.7) | 58.4 (52.2, 61.5) |
| Threonine | 123 (114, 132) | 131 (119, 136) | 133 (126, 141) | 120 (115, 126) |
| Tryptophan | 26.4 (24.8, 30.2) | 28.3 (23, 32.6) | 31.2 (29.6, 32.3) | 30.5 (29.6, 31.1) |
| Tyrosine | 81 (73.7, 84.3) | 67.3 (58.3, 76.9) | 70.4 (64.6, 76.4) | 75.3 (70, 83.1) |
| Valine | 275 (264, 301) | 281 (280, 290) | 300 (289, 321) | 301 (300, 306) |
| 3-methyl-histidine | 4.32 (2.79, 6.73) | 4.17 (2.39, 6.06) | 9.93 (5.02, 16) | 5.29 (2.29, 8.09) |

| **Supplemental Table S2.** Mean (standard deviation) urinary excretion of amino acids according to mesna dosing | | | | | | |
| --- | --- | --- | --- | --- | --- | --- |
|  | **400 mg** | **800 mg** | **1200 mg** | **1600 mg** | **β (95 % CI)** | **p** |
| *Amino acid, μmol/24 h* | | | | | | |
| Alanine | 164 (49.3) | 90.1 (47.3) | 270 (141) | 135 (60.4) | 8.81 (-28.9, 46.5) | 0.63 |
| Arginine | 16.2 (7.99) | 11.9 (6.25) | 27.3 (14.7) | 17.5 (14) | 1.90 (-2.46, 6.26) | 0.38 |
| Asparagine | 36.4 (15.1) | 20.5 (11.7) | 84.4 (70.3) | 36.9 (12.8) | 6.47 (-8.74, 21.7) | 0.39 |
| Glutamate | 16.4 (12.6) | 5.68 (2.91) | 14.7 (8.3) | 10.9 (8.18) | -0.91 (-4.38, 2.56) | 0.59 |
| Glutamine | 326 (142) | 170 (53.6) | 459 (273) | 280 (127) | 13.4 (-56.3, 83.0) | 0.70 |
| Glycine | 1090 (663) | 543 (202) | 1150 (387) | 682 (244) | -68.2 (-245, 109) | 0.43 |
| Histidine | 561 (143) | 330 (96.7) | 856 (602) | 457 (89.3) | 19.6 (-110, 150) | 0.76 |
| Isoleucine | 7.38 (4.47) | 3.28 (1.83) | 10.1 (7.83) | 5 (3.47) | -0.07 (-2.03, 1.88) | 0.94 |
| Leucine | 20.6 (11.1) | 10.8 (4.02) | 27.4 (20.1) | 15.7 (7.22) | 0.09 (-4.72, 4.91) | 0.97 |
| Lysine | 81.6 (45.8) | 45.3 (33.3) | 103 (100) | 47.9 (27.3) | -4.62 (-26.8, 17.6) | 0.67 |
| Ornithine | 11.8 (6.47) | 6.87 (2.99) | 12.6 (5.67) | 9.65 (3.24) | -0.13 (-2.03, 1.78) | 0.89 |
| Phenylalanine | 42.8 (24.3) | 18.7 (9.08) | 56.9 (46.5) | 31.8 (12.5) | 0.27 (-10.6, 11.1) | 0.96 |
| Proline | 2.35 (1.37) | 0.51 (0.178) | 3.78 (3.31) | 1.25 (0.283) | 0.05 (-0.85, 0.95) | 0.91 |
| Serine | 223 (116) | 129 (34) | 276 (96.9) | 169 (36.7) | -2.58 (-37.7, 32.6) | 0.88 |
| Taurine | 271 (320) | 379 (262) | 482 (423) | 346 (520) | 35.0 (-103, 173) | 0.61 |
| Threonine | 78.3 (27) | 43.2 (14.2) | 115 (68.5) | 58.1 (20.1) | 0.78 (-15.9, 17.5) | 0.92 |
| Tryptophan | 25 (11.4) | 13.4 (5.04) | 41.4 (36.5) | 21 (6.46) | 1.52 (-6.16, 9.2) | 0.69 |
| Tyrosine | 64.7 (26) | 33.5 (14.5) | 104 (94.8) | 53.6 (17.2) | 3.41 (-16.3, 23.1) | 0.72 |
| Valine | 22.6 (16.9) | 10.3 (7.58) | 32.7 (27.5) | 17.2 (12.7) | 0.34 (-6.67, 7.34) | 0.92 |
| 3-methyl-histidine | 558 (261) | 234 (189) | 503 (270) | 327 (219) | -46.3 (-141, 48.5) | 0.32 |
